# Predictors of Mortality Amongst Tocilizumab Administered COVID-19 Asian Indians: A Predictive Study From a Tertiary Care Centre

**DOI:** 10.7759/cureus.13116

**Published:** 2021-02-04

**Authors:** Hardik D Desai, Kamal Sharma, Atul Parikh, Karan Patel, Jayesh Trivedi, Rupak Desai, Parth P Patel, Zeel Patel, Smeet Patel, Saurav Kini

**Affiliations:** 1 Graduate Medical Education, Gujarat Adani Institute of Medical Sciences, Bhuj, IND; 2 Department of Cardiology, U N Mehta Institute of Cardiology and Research Centre, Ahmedabad, IND; 3 Department of Medicine, Shah Alloys Limited (SAL) Hospital and Medical Institute, Ahmedabad, IND; 4 Department of Cardiology, Atlanta Veterans Affairs Medical Center, Decatur, USA; 5 Graduate Medical Education, Ahmedabad Municipal Corporation Medical Education Trust (AMC MET) Medical College, Ahmedabad, IND; 6 Graduate Medical Education, Smt. Nathiba Hargovandas Lakhmichand (N.H.L) Municipal Medical College, Ahmedabad, IND

**Keywords:** covid-19, sars-cov-2, tocilizumab, predictors of mortality

## Abstract

Introduction

Hyper-cytokinemia is a dreaded complication of severe acute respiratory syndrome coronavirus 2 (SARS-Cov-2) infection and an important predictor of mortality in coronavirus disease 2019 (COVID-19). The current evidence at best is still ambiguous for use of tocilizumab in cytokine storm in COVID-19. Moreover, the factors that are associated with beneficial response from tocilizumab are unknown in COVID-19. We aimed to study the clinical outcomes especially mortality vis-à-vis clinical and laboratory characteristics of patients administered tocilizumab and identify predictors of mortality benefits amongst deceased vs recovered COVID-19 patients.

Methods

The present study is a retrospective observation of the demographic, clinical, and biological data of all the consecutive patients treated with tocilizumab for COVID-19 pneumonia at the COVID tertiary care centre from July 2020 to October 2020 at Ahmedabad, India. We compared the deceased group with those who recovered/discharged and evaluated patient-level demographics, clinical attributes, and laboratory investigations available to identify subgroups in whom tocilizumab reduced mortality.

Results

Of the 112 patients included, the mean (SD) age was 56.84 ± 13.56 years and 80 (71.4%) were male. There were 97 (86.6%) patients in the survivors and 15 (13.39%) in the deceased group. Deceased were older than the recovered group (mean: 66.14, SD: 14.41 vs mean: 55.36, SD: 12.98; p=0.04). Hypertension (33.03%) was the commonest comorbidity observed. Mortality was significantly higher in patients with cancer and type-2 diabetes (p=0.05 and p=0.01, respectively). Level of D-dimer and lactate dehydrogenase (LDH) showed trends towards significance as a predictor of mortality (p=0.07 and p=0.08, respectively) not reaching significance. D‐dimer level > 5,000 nanograms per millilitre (ng/mL) was the significant predictor of subsequent deaths (p<0.0001). Fourteen patients reported adverse events of tocilizumab. Patients who developed in-hospital complications (such as septic or vasodilatory shock and/or sepsis, acute kidney injury, multiorgan dysfunction) had significantly higher mortality (p<0.0001, p=0.009, and p=0.03, respectively).

Conclusion

Tocilizumab might be more beneficial in younger patients without sepsis/ septic shock, acute kidney injury, multiorgan dysfunction, and who were non-ventilated. The predictors of mortality amongst Asian Indians treated with tocilizumab were older patients, the presence of type-2 diabetes, cancer, in-hospital complication (such as acute kidney injury, sepsis/septic shock, multiorgan dysfunction), higher D-dimer > 5,000 ng/mL. A larger study with pre-defined inclusion cut-offs of these variables may aid in defining patient's characteristics of Asian Indians who may benefit from tocilizumab in COVID-19.

## Introduction

Since December 2019, coronavirus disease 2019 (COVID-19) has affected 21,026,758 people and 755,786 death worldwide with a case fatality rate (CFR) of 3.59% as per the World Health Organization (WHO) situation report as of December 31, 2020. Current evidence suggests that refractory cases of COVID-19 have poorer outcomes due to cytokine storm or cytokine releasing syndrome (CRS), characterized by elevation of inflammatory cytokines such as interleukin-6 (IL-6), IL-2, IL-7, TNF-A, IL-1 B, IL-1 Ra, IL-10, IP-10, granulocyte-colony stimulating factor, monocyte chemoattractant protein and is associated with fatal outcome in COVID-19. These patients usually have abnormal inflammatory markers including elevated serum levels of IL-6, ferritin, and C-reactive protein (CRP). These inflammatory markers help to prognosticate and define the disease severity [1-2]. Histology autopsy findings of samples collected from patients that had died with COVID-19 showed bilateral diffuse alveolar injury accompanied by cell-fibromyxoid exudates with inflammatory interstitial mononuclear cell infiltration, primarily lymphocytes [3]. It has been suggested that therapeutic interventions that dampen inflammatory cytokine production may alleviate inflammation and decrease mortality in COVID-19, given the histopathological findings in CRS in critical and lethal COVID-19.

Elevated IL-6 is a prominent and constitutive factor of CRS, just like CRS in severe acute respiratory syndrome (SARS) or Middle East respiratory syndrome (MERS) patients [4]. With respect to the cytokine storm in patients with COVID-19, Zhou et al. [5] had stated that serum IL-6 levels were increased in patients with COVID-19 (n=33) due to a significantly higher proportion of CD14+, CD16+, and inflammatory monocytes. Higher serum IL-6 concentrations correlated with higher SARS Coronavirus-2 (SARS-CoV-2) viremia levels, sustained viral ribonucleic acid (RNA) shedding, progression to mechanical ventilation, and death [6-7]. These results lead us to hypothesize that this inflammatory pathway could be halted at a critical point by using the interleukin-6 receptor (IL-6R) blockade. Tocilizumab is a humanized monoclonal antibody against the IL-6R and is a Food and Drug Administration (FDA) approved drug for the treatment of rheumatoid arthritis (RA), systemic juvenile idiopathic arthritis, giant cell arteritis (GCA), and CRS. There have been contrary findings from various nonrandomized and open-label studies with evidence from randomized, double-blind, placebo-controlled trials not being so robust [8-19]. Results from non-peer-reviewed publications have demonstrated that two immunosuppressive IL-6 inhibitors, aimed at abrogating, or at least mitigating cytokine storms - viz tocilizumab and sarilumab, have been found to reduce mortality by 8.5% amongst critically ill patients including those on ventilators [20]. Furthermore, the factors that are associated with beneficial response from tocilizumab are unknown in COVID-19. Asian Indians have a substantial prevalence and disease burden. Asian Indians also have a higher prevalence of diabetes, hypertension, and cardiac disease as compared to any other ethnicity. Barring a couple of case series and small observational data, there is a paucity of literature about the efficacy of tocilizumab amongst Asian Indians despite its high usage in CRS in the current pandemic. We aimed to study the clinical outcomes especially mortality vis-à-vis clinical and laboratory characteristics of patients administered tocilizumab and identify predictors of mortality benefits amongst deceased vs recovered COVID-19 patients.

## Materials and methods

This is a retrospective observational study of the demographic, clinical, and biological data of all the consecutive patients treated with tocilizumab for COVID-19 pneumonia at the COVID tertiary care centre from July 2020 to October 2020 at Ahmedabad, India.

Study population

Inclusion criteria were (1) age > 18 years, (2) all severity levels of COVID-19 who were administered tocilizumab during hospitalization, and (3) Asian Indians with SARS-CoV-2 infection confirmed by a real-time polymerase chain reaction (RT-PCR). All the eligible patients had at least one of the serological criteria including CRP level > 50 milligram per litre (mg/L), serum ferritin level > 500 nanogram/millilitre (ng/mL), D-dimer level > 1,000 ng/mL, or a lactate dehydrogenase level (LDH) > 250 unit per liter (U/L). Exclusion criteria for the study were: (1) age < 18 years, (2) prior organ transplant recipient, (3) recurrent COVID-19 infection, (4) pregnant patient with COVID-19, (5) prior treatment with immunosuppressive or biological agents during hospital course, and (6) history of severe allergic reactions to monoclonal antibodies.

Procedure - treatment and data collection

All the patients were treated as per the standard protocol of hospital guidelines for COVID-19 (included oxygen supply to maintain oxygen saturation (SaO_2_) reaching at least > 90%, injection remdesivir 200 mg on day one followed by 100 mg for the next five days, azithromycin (500 mg once per day for five days), and low molecular weight heparin (LMWH) for prophylaxis of thrombosis according to bodyweight and renal clearance). Tocilizumab was administered by the intravenous route at 8 milligram/kilogram (mg/kg) body weight (up to a maximum of 800 mg) administered once or twice, 12 hours apart. The second dose was administered to attain adequate plasma concentration of the drug based on the findings of pharmacokinetic models for severe or life-threatening T cell-induced cytokine storm in adult and paediatric patients [21]. Extracted data included age, gender, baseline clinical and laboratory characteristics, and the outcomes with a focus on mortality and discharges, the severity of the disease, tocilizumab adverse event, in-hospital complications, duration of symptom onset to hospital admission, day of hospitalization for all tocilizumab administered patients. All patients treated with tocilizumab for COVID-19 were classified into two groups: deceased and recovery. We compared baseline demographics, clinical and laboratory characteristics in both groups.

Statistical analysis

On the collected data, a descriptive analysis was conducted. Continuous variables were summarized as mean (and/or range), whereas categorical variables were presented as number (percentage) and range. Comparison between “Deceased vs Recovered” was performed using Chi-square test or Fisher’s exact test, and Student’s t-test or Wilcoxon-Mann-Whitney test. A single primary analysis with the statistical significance criterion specified as a two-sided p-value of less than 0.05 was performed using Statistical Package for the Social Sciences (SPSS) software version 22 (IBM Corp., Armonk, NY).

## Results

Patients' characteristics and clinical outcomes

From a total cohort of 1,324 patients hospitalized for COVID-19, 112 patients had received tocilizumab at the hospital apart from the “standard care" for COVID-19 management. The mean age of these 112 Asian Indians who received tocilizumab was 56.84 years (SD: 13.56, range: 26-88) amongst them 80 were male and 32 were female. There were a broad range of comorbidities reported such as hypertension, diabetes, myocarditis, asthma or chronic obstructive pulmonary disease (COPD), gastrointestinal reflux disease (GERD), migraine, dementia, Parkinson's disease, chronic kidney disease (CKD) or end-stage renal disorders (ESRD), arthritis, lipid disorders, obesity, anaemia, and malignancy. Hypertension 37/112 (33.03%) was the commonest comorbidity. Mortality amongst patients receiving tocilizumab was significantly higher in patients with cancer and type-2 diabetes (p=0.05 and p=0.01, respectively). Patients presented with various signs and symptoms such as fever, cough, diarrhoea, headache, myalgia, fatigue, chest tightness, chest pain, nausea/vomiting, sore throat, tachypnoea, dyspnoea, and tachycardia. In-hospital reported complications were acute respiratory distress syndrome (ARDS), septic or vasodilatory shock and/or sepsis, cardiomyopathy (such as takotsubo cardiomyopathy, hyper-obstructive cardiomyopathy), pneumonia, and acute kidney injury (AKI). Fifteen out of 112 (13.39%) died and 97 (86.60%) were discharged alive. One remained hospitalized due to ongoing pulmonary (comorbidity) issues and three required treatments for fungal infection reported as a tocilizumab adverse event.

Laboratory characteristics and in-hospital management

There was no significant difference for total days from symptoms onset to hospital admission in deceased vs hospitalized group or gender differences between male vs female. The majority of patients, 89 out of 112 (79.46%), were given tocilizumab within seven days of hospitalization. Most of the patients reported clinical improvement and/or laboratory improvement within one to two days. Ninety four out of 112 (83.9%) reported an elevated LDH > 300 U/L. Sixteen out of 112 (14.28%) reported D-dimer level > 5,000 ng/mL. D‐dimer level > 5,000 ng/mL was the significant predictor of subsequent deaths amongst Asian Indians receiving tocilizumab in the cohort (p<0.0001). Seventy-four out of 112 (66.06%) reported an elevated ferritin level > 1,000 ng/mL of which seven reported > 3,000 ng/mL. Sixty-eight patients reported an elevated IL-6. Ninety five out of 112 (84.8%) had an elevated CRP level > 100 mg/L. Consolidation 12/15 (80%) was frequently observed in chest imaging amongst the deceased group. Ninety three out of 112 (83.03%) had required either non-invasive ventilation (NIV) or invasive ventilation. However, there is no significant difference in mortality observed between ventilation requiring patients compared to those who did not require it, although mortality in the tocilizumab cohort was significantly higher in patients requiring NIV (p=0.04). All 11 patients with moderate and/or moderate-severe conditions recovered. There is no significant difference observed between the deceased group and the recovered group amongst the severe/critical vs moderate/severe-moderate. However, there was a trend towards higher mortality in patients with severe disease (p=0.06) (Table 1).

**Table 1:** Baseline clinical and laboratory characteristics and outcomes in COVID-19 patients treated with tocilizumab SD: standard deviation, CRP: C-reactive protein, IL-6: interleukin-6, LDH: lactate dehydrogenase, CXR: chest X-ray, CT: computed tomography, HRCT: high- resolution CT, HF: high flow, NIV: non-invasive ventilation, ARDS: acute respiratory distress syndrome, AKI: acute kidney injury, CNS: central nervous system, GGO: ground-glass opacity

Variable	Recovered and Discharged/Hospitalized (N: 97) Mean or n (Range or %) (n)	Death (N: 15) Mean or n (Range or %) (n)	P-value	Total (N:112) Mean or n (Range or %) (n)
Age (Years) Mean ± SD (Range)	55.36 ± 12.98 (26-78)	66.14 ± 14.41 (40-88)	0.049	56.84 ± 13.56 (26-88)
Male (n)	70 (72.16%)	10 (66.6%)	0.66	80 (71.42%)
Female (n)	27 (27.83%)	5 (33.33%)		32 (28.57%)
Comorbidities				
Hypertension	33 (34.02%)	4 (26.66%)	0.57	37 (33.03%)
Type 2 Diabetes	13 (13.40%)	6 (40%)	0.01	19 (16.96%)
Cardiac	11 (11.34%)	2 (13.33%)	0.82	13 (11.60%)
Respiratory	13 (13.40%)	0	0.27	13 (11.60%)
CNS	7 (7.21%)	2 (13.33%)	0.42	9 (8.03%)
Renal	4 (4.12%)	2 (13.33%)	0.16	6 (5.88%)
Gastrointestinal	7 (7.21%)	0	0.52	7 (5.35%)
Rheumatology/Metabolic/Infectious	18 (18.55%)	4 (26.66%)	0.46	22 (19.64%)
Haematology	4 (4.12%)	2 (13.33%)	0.16	6 (5.35%)
Cancer	2 (2.06%)	2 (13.33%)	0.05	4 (3.57%)
Psychiatry	2 (2.06%)	0	0.89	2 (1.78%)
Laboratory characteristics and chest Imaging				
D-dimer (ng/mL)	2,866.27 (310-9,700)	5,794.2 (1,100-10,990)	0.07	3,387.04 (310-10,990)
D-dimer < 5,000 (ng/mL) (n)	93 (95.87%)	03 (20%)	<0.0001	96 (85.71%)
D-dimer > 5,000 (ng/mL) (n)	04 (4.12%)	12 (80%)		16 (14.28%)
CRP (mg/L)	241.20 (5.09-2,386)	91.56 (9-179)	0.67	214.48 (5.09-2,386)
Ferritin (ng/mL)	1,266.45 (108-6,045.6)	1,830.4 (376-3,850)	0.43	1,389.05 (108-6,045.6)
IL-6 (pg/mL)	176 (32-1,040)	104.65 (74.3-135)	0.69	168 (32-1,040)
LDH (U/L)	373.33 (185-890)	573 (377-802)	0.08	401.85 (185-890)
CXR	58 (59.79%)	7 (46.6%)	-	65/112 (58.03)
Patchy Infiltrates	46/58 (79.31%)	03/07 (42.85%)	-	49/65 (75.38%)
Consolidation	12/58 (20.68%)	04/07 (57.14%)	-	16/65 (24.61%)
CT	36 (37.11%)	0	-	36/112 (32.14)
GGO	26/36 (72.22%)	0	-	26/36 (72.22%)
Consolidation	10/36 (27.77%)	0	-	b. 10/36 (27.77%)
HR-CT	03 (3.09%)	08 (53.33%)	-	11/112 (9.82%)
GGO	02/03 (66.66%)	0		02/11 (18.18%)
Consolidation	01/03 (33.33%)	08 (100%)		09/11 (81.81%)
Oxygen Requirement				
Nasal Canula	6 (6.18%)	0	0.59	6 (5.35%)
Mask/HF	13 (13.40%)	0	0.27	13 (11.60%)
NIV	16 (16.49%)	6 (40%)	0.04	22 (19.64%)
Invasive Ventilation	62 (63.91%)	9 (60%)	0.76	71 (63.39%)
In-Hospital Complication				
ARDS	68 (70.10%)	12 (80%)	0.43	80 (71.42%)
Pneumonia	18 (18.55%)	0	0.14	18 (16.07%)
Septic Shock or Sepsis	3 (3.09%)	9 (60%)	<0.0001	12 (10.71%)
AKI	0	3 (20%)	0.009	3 (2.62%)
Multiorgan Dysfunction	4 (4.12%)	3 (20%)	0.03	7 (6.25%)
Cardiomyopathy	3 (3.09%)	0	0.92	3 (2.67%)
Anemia	1 (1.03%)	1 (20%)	0.18	2 (1.78%)
Outcome and other characteristics				
Symptoms Onset to Hospital Admission (Days)	7.3 (3-14)	6.8 (3-10)	0.76	7.22 (3-14)
Hospital Admission to Tocilizumab Administration (Days)	5.46 (1-18)	4.33 (2-13)	0.48	5.32 (1-18)
When Improvement Noticed After Tocilizumab (Days)	1.53 (0.5-4)	1.75 (0.5-3)	0.71	1.54 (0.5-4)
Total Length of Hospital Stay After Tocilizumab (Days)	9.96 (3-20)	10 (1-32)	0.98	9.97 (1-32)
Disease Severity				
Moderate	3 (3.09%)	0	0.92	3 (2.67%)
Severe	16 (16.49%)	6 (40%)	0.06	22 (19.64%)
Critical	70 (72.16%)	9 (60%)	0.34	79 (70.73%)
Moderate-Severe	8 (8.24%)	0	0.46	8 (7.14)

Adverse events of tocilizumab

A total of 14 patients reported adverse events such as drug-induced liver injury or abnormal liver profile, bacterial or fungal infection, transaminitis, and hypertriglyceridemia.

## Discussion

Hyper-cytokinemia is a hallmark of SARS-CoV-2 usually developing in the second week during COVID-19 is associated with a state of both immunodeficiency and hyper inflammation, with the latter being manifested as cytokine storm [22]. The elevated cytokine levels may also be responsible for the lethal complications of COVID-19 which are significantly associated with increased mortality. In particular, this research offers some useful details on the profile of patients who may or may not benefit from treatment with IL-6 blockade with tocilizumab amongst the Asian Indian cohort. We observed several demographic and clinical characteristics that were more common in the deceased group. Mortality was significantly higher in the older age group, which was consistent with the other studies [23]. Elderly patients encountered higher age-related changes that may have impaired their ability to respond appropriately to pathogens with an effective immune response. The development of new naive T cells declines dramatically between 40 and 50 years of age, impairing these older adults' ability to combat new viruses such as SARS-CoV-2 [24]. As age advances, the prevalence of comorbidities such as diabetes mellitus and hypertension increases. In the present study, overall hypertension was the commonest comorbidity observed whereas, diabetes was significantly more common in the deceased group (p=0.01). One study has also reported that tocilizumab in hyperglycemic patients, failed to stop the progression of risk of severe outcomes as compared to those in the normoglycemic patients (p≤0.009) [25]. While it has been shown that cardiac disease, bone disease, and malignancy raise the likelihood of poorer clinical outcomes in SARS-CoV-2 infections, further studies are required to examine the mechanisms observed in COVID-19 in particular [26]. Furthermore, higher D-dimer and LDH levels had trends to be associated with mortality but did not reach statistical significance (p=0.07 and p=0.08, respectively) and are congruence with the findings of other studies, showing that D-dimer was observed to be higher in deceased patients compared to survivors amongst those treated with tocilizumab but the substantial correlation is still missing [27].

We have observed that patients with D-dimer > 5,000 (ng/mL) had significantly higher mortality than those with < 5,000 (ng/mL) (p<0.0001). Lippi et al. have also reported that elevated D-dimer in COVID-19 patients is associated with higher mortality [28]. This study warrants the use of tocilizumab in patients early that is when administered at lower values of D-dimer as compared to those with higher D-dimer levels as much as >4,000 ng/mL. Eleven patients reported persistent or new elevation of D-dimer level even after administration of tocilizumab. One cohort study has reported that tocilizumab decreases factor XIII level which may stabilize the fibrin [29]. These findings warrant the administration of anticoagulants in COVID-19 patients who are treated with tocilizumab. Thirty-two patients reported an elevated IL-6 level while three reported gradually decreased levels after the first dose of tocilizumab. An initial rise of IL-6 level and then gradually decreased is probably explained by its pharmacokinetic mechanism. In the present study, tocilizumab was well tolerated, except for 14 patients. Four patients developed a drug-induced liver injury or abnormal liver profile and eight patients developed bacterial and/or fungal infection. Another patient had cardiac complications during administration. However, previous studies have demonstrated that tocilizumab may have a cardioprotective effect as it reduces corrected QT interval apart from reducing the inflammatory mediators which may contribute to the progression of myocardial damage [30]. One patient developed transaminitis and hypertriglyceridemia. Clinicians should use tocilizumab with caution in patients with associated risk factors and monitor these high-risk patients more closely. We have observed in-hospital medical illnesses such as ARDS, pneumonia, and AKI in the analysis. Twelve (10.71%) patients developed septic or vasodilatory shock and/or sepsis of which nine died. We observed that patients who developed shock, AKI, and multiorgan dysfunction as in-hospital complications had higher mortality and it was statistically significant (p<0.0001, p=0.009, and p=0.03, respectively).

The results show that the majority of patients required NIV or invasive ventilation during hospitalization which is in line with most of the institutional guidelines where tocilizumab is usually administered at later or advanced stages of illnesses. All 12 patients treated with tocilizumab during moderate and/or moderate-severe stage of clinical illness recovered completely. In the cohort, all those patients recovered who did not require mechanical ventilation while in contrast, 15/93 (16.12%) patients who required mechanical ventilation died. However, there is no significant association for mortality between those who required mechanical ventilation vs those who did not. Our results are consistent with a retrospective cohort study done by Guaraldi et al. which shows similar outcomes of tocilizumab treatment in mechanically ventilated COVID-19 patients in terms of mortality (20.83% vs 14.28%) [8].

The results of this study are encouraging (86.6% improved and/or discharged, remained stable or with moderate disease with tocilizumab), but should be evaluated with caution due to the relatively small sample size. Due to the retrospective nature of the study, the findings may be confounded by bias, including variations in baseline characteristics and duration of stay. Additionally, the aforementioned positive findings could be misleading because most of the patients were seen within a brief period of time, perceived by the physician as the best "opportunity timeframe" for using tocilizumab. The dosage of tocilizumab used and its application at the required time during the course of the disease was left at the discretion of the treating intensivist and could have caused systemic bias. While randomized controlled clinical trials are needed to demonstrate the effectiveness of tocilizumab per se, more investigations are required to clarify any possible differences in terms of efficacy and adverse effects between moderate COVID-19 vs severely/critically affected-ventilation requiring COVID-19 patients, especially amongst Asian Indians.

## Conclusions

The present study shows an 86.6% recovery rate in patients treated with tocilizumab thus, positioning it as a potentially promising therapy in COVID-19 Asian Indians especially with moderate/severe disease. It might be more beneficial in non-ventilated or moderate/moderate-severely affected COVID-19 patients. The predictors of mortality amongst Asian Indians treated with tocilizumab were older patients, presence of type-2 diabetes, cancer, in-hospital complication (such as AKI, sepsis/septic shock, multiorgan dysfunction), and higher D-dimer > 5,000 ng/mL. There was a trend towards higher mortality in patients with severe COVID-19, higher LDH, and D-dimer. A larger study with pre-defined inclusion cut-offs of these variables may aid in defining patient characteristics of Asian Indians who may benefit from tocilizumab in COVID-19. These findings need further review in clinical trials, owing to the retrospective aspect of our research, to establish the role of IL-6 blockade, its benefit, dosing, and the timing of administration in moderate to critically ill COVID-19 patients.
